# Yoga-based meditation improves self-regulation and well-being in medical students: a pilot study

**DOI:** 10.3389/fnhum.2026.1844429

**Published:** 2026-08-12

**Authors:** Felipe Rojas-Thomas, Ignacia Cantwell, Claudio Artigas, Diego Garrido, Jessica Goset, María Jose Olivares, Joaquín Araya, Benjamin Ibañez, Antonella Pereira, María Carolina Otero, Luis Sebastián Contreras-Huerta, Ricardo Ramirez-Barrantes

**Affiliations:** 1 Center for Social and Cognitive Neuroscience (CSCN), School of Psychology, Universidad Adolfo Ibáñez, Santiago, Chile; 2 Laboratory of Neuroeducation and Well-Being, Faculty of Medicine, Andrés Bello University, Viña del Mar, Chile; 3 Biology Department, Universidad Autónoma de Chile, Temuco, Chile; 4 Escuela de Tecnología Médica, Facultad de Salud, Universidad Santo Tomás, Santiago, Chile; 5 Centro de Investigación en Prevención y Cuidados de la Salud (+SALUD), Facultad de Salud, Universidad Santo Tomás, Santiago, Chile; 6 School of Chemistry and Pharmacy, Faculty of Medicine, Andrés Bello University, Santiago, Chile; 7 School of Medical Technology, Faculty of Medicine, Andrés Bello University, Viña del Mar, Chile

**Keywords:** cognitive functions, meditation, self-regulation, well-being, yoga

## Abstract

**Introduction:**

Self-regulation enables students to effectively apply their metacognitive abilities to reach academic objectives and enhance their well-being, which is essential in academic high-pressure situations such as medical education. However, many medical students have difficulty managing their cognitive and emotional responses to continued academic pressure and the clinical demands placed upon them. Thus, it is important to identify methods that may support the self-regulatory abilities of medical students.

**Methods:**

This study assessed whether participation in a yoga-based meditation program was associated with pre-to-post changes in cognitive and psychological indicators of self-regulation among medical students. The sample consisted of forty subjects who completed a series of self-report questionnaires assessing anxiety, stress, depression (DASS-21 and STAI), mindfulness (FFMQ), and overall mental health or psychological well-being (WEMWBS). In addition to completing the self-report measures, each subject’s cognitive performance was measured before and after completion of the intervention program using three separate tasks: AOSPAN for working memory, a free recall task to measure episodic memory, and SART for sustained attention.

**Results:**

Pre/post assessments revealed statistically significant decreases in reported levels of anxiety, stress, and depression. Conversely, there were statistically significant increases in reported levels of psychological well-being and global mindfulness meta-awareness. Following the intervention, cognitive task performance also changed. Specifically, we observed significant increases in working memory performance, immediate and delayed episodic memory, and response accuracy, together with response-time dynamics consistent with a more cautious response pattern.

**Discussion:**

These results suggest that yoga-based meditation was associated with pre-to-post changes in cognitive and psychological indicators of self-regulation, although causal interpretation is limited by the single-group pilot design. Taken together, these findings support further investigation of meditation-based programs as low-cost and scalable approaches to support cognitive performance and overall well-being in demanding educational settings such as medical education.

## 1 Introduction

Self-regulation is a central cognitive resource for adaptive thought, purposeful action, and psychological health. It involves the capacity to monitor one’s mental states, emotions, and actions, and to adjust them according to situational demands or long-term goals (Inzlicht et al., 2021). This capacity depends in part on executive functions, including working memory, inhibitory control, and cognitive flexibility, which help maintain goal-relevant information, suppress competing impulses, and organize behavior over time (Diamond, 2020; Salehinejad et al., 2021). At a neurocognitive level, these processes rely on interactions between prefrontal control systems and broader networks involved in attention, motivation, and behavioral regulation (Hofmann et al., 2012; Menon, 2011).

Because attention is one of the main routes through which self-regulation operates, meditation has been increasingly studied as a form of mental training. In focused-attention practices, such as attending to the breath or bodily sensations, practitioners learn to notice when attention has drifted and to return it to the chosen object (Lutz et al., 2008; Tang et al., 2015). This repeated process may support attentional monitoring and reduce the impact of mind-wandering on ongoing performance (Feruglio et al., 2021; Sim et al., 2024; Smallwood and Schooler, 2015). Neuroimaging studies also suggest that meditation is associated with changes in prefrontal and default mode regions involved in cognitive control and internally oriented processing, although these neural findings vary across practices and populations (Calderone et al., 2024; Sezer et al., 2022). In behavioral terms, meditation has also been associated with improvements in working memory and related cognitive abilities, but effect sizes appear to depend on the type of practice, training duration, and participant characteristics (Tang et al., 2007; Tang et al., 2015).

Yoga-based meditation may be particularly relevant for self-regulation because yoga traditions train relaxation, regulation of attention, bodily awareness, and regulation of mental activity within an integrated training system. This is not identical to standardized mindfulness-based programs such as Mindfulness-Based Stress Reduction (MBSR), which are usually structured as clinical or psychoeducational interventions and often include group discussion, stress education, and home assignments. From a mechanistic perspective, yoga-based meditation may engage both top-down processes, such as attentional control, executive monitoring, and meta-awareness, and bottom-up processes related to interoceptive, autonomic, and bodily feedback (Dahl et al., 2015; Gard et al., 2014; Lutz et al., 2008; Schmalzl et al., 2015; Tang et al., 2015). This makes it a useful contemplative approach for examining how attentional training and bodily regulation may jointly contribute to self-regulation.

These mechanisms are especially relevant in medical education. Medical students are exposed to prolonged academic pressure, heavy cognitive workloads, emotionally demanding learning environments, and variable levels of stress throughout training. Psychological distress has been reported around examinations and at the end of academic semesters, often accompanied by anxiety, emotional exhaustion, and poor sleep quality (Aljuwaiser et al., 2024; Almutairi et al., 2022; Carrard et al., 2024; Cotobal Rodeles et al., 2025; Mazzoleni et al., 2024; Mohmand et al., 2022). This burden may begin during medical training itself: a large systematic review and meta-analysis found that depressive symptoms increased after students entered medical school, with a median absolute increase of 13.5% across longitudinal studies (Rotenstein et al., 2016). More recent reviews suggest that the problem may have intensified after the COVID-19 pandemic, with high levels of depression, anxiety, stress, sleep disturbance, and emotional exhaustion reported among medical students (Jia et al., 2022; Lin et al., 2024; Peng et al., 2022). Taken together, these findings suggest that psychological distress in medical students should not be viewed as an isolated or transient event, but as a persistent and possibly increasing challenge in medical education, particularly under conditions of academic pressure, insufficient sleep, limited social interaction, and uncertainty about training pathways (Aljuwaiser et al., 2024; Lin et al., 2024; Peng et al., 2022).

**Figure 1:**
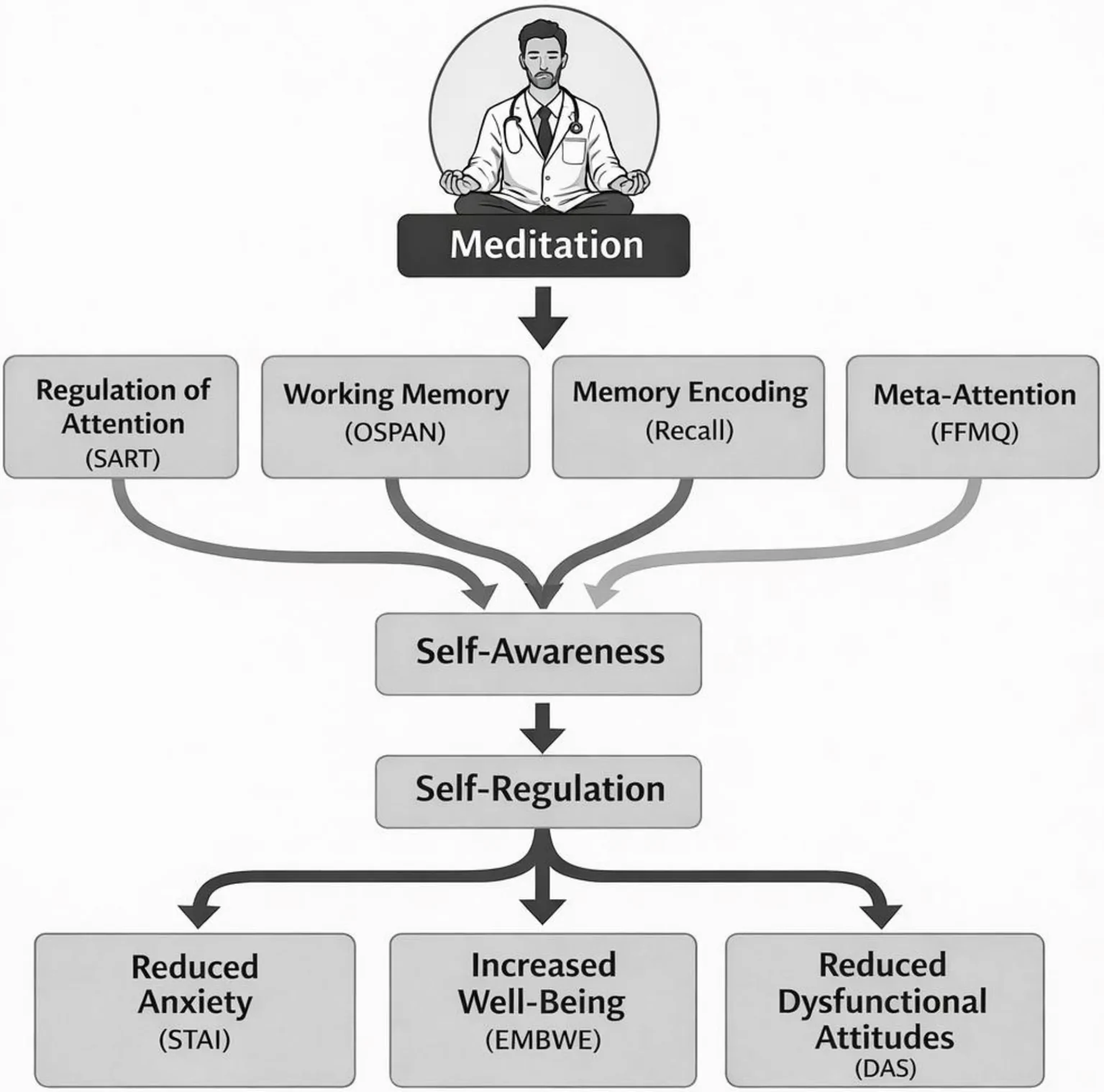
Conceptual model of the proposed associations between meditation and self-regulation in medical students. Meditation is proposed to engage four cognitive domains relevant to self-regulation: regulation of attention (assessed via SART), working memory (AOSPAN), memory encoding (free recall), and meta-attention (FFMQ). Collectively, these processes may contribute to self-awareness, which in turn supports self-regulation. Downstream psychological indicators include reduced anxiety, depression, and stress (STAI and DASS-21), and increased well-being (WEMWBS). This framework is grounded in the S-ART model (Vago and Silbersweig, 2012) and provides the theoretical basis for evaluating whether a yoga-based meditation program may be associated with self-regulatory capacity and psychological well-being in medical students.

Mindfulness-based programs have shown benefits for emotional dysregulation and stress in medical students (Hathaisaard et al., 2022; Kaisti et al., 2023). However, mindfulness represents only one part of the broader field of contemplative interventions. In the present pilot study, we examined a yoga-based meditation program focused on progressive attentional training and assessed its association with psychological health and cognitive indicators of self-regulation. We hypothesized that participation in this program would be associated with pre-to-post changes in multidimensional markers of self-regulation, including attentional control, working memory, episodic memory performance, mindfulness-related meta-awareness, and psychological well-being (Figure 1).

## 2 Methods

*Study Design*: The study employed a quasi-experimental single-group pre-post design to examine changes in cognitive and psychological indicators of self-regulation following a meditation intervention in medical students. The primary dependent variables included performance on several cognitive tests and completion of a series of standardized self-report surveys designed to measure various domains, including cognitive functions (executive functioning, sustained attention, and working memory), affective states (stress, anxiety, and depression), and well-being-related constructs (mindfulness traits and overall psychological well-being). Both sets of data were collected at roughly the same point in time, about 6 weeks apart.

At Time 1 (week one) all participants completed their baseline measurements which consisted of completing a series of cognitive tasks and completing a series of standardized self-report survey instruments. After completion of the baseline assessments participants received instruction for participation in a four-week long meditation training program. Approximately 1 week after completion of the meditation program (Time 2), participants completed post-intervention assessments with respect to the same cognitive and psychological survey measures.

*Subjects*: A total of 40 undergraduate medical students voluntarily participated in the study. Subjects were solicited from undergraduate medical education programs at each university involved in this collaborative research effort via institutional public announcement and volunteer registration. Participation eligibility requirements included: being currently enrolled in an undergraduate medical education program; being between 18 and 35 years old, an age range selected to obtain a relatively homogeneous undergraduate student sample and reduce potential age-related variability in cognitive performance and psychological outcomes (Hartshorne and Germine, 2015); being willing to commit to a 4-week period of regular meditation training; and having sufficient time available to attend scheduled training sessions and complete both pre- and post-intervention assessments. Exclusionary criteria included: having previously formally trained in meditation practices greater than six months; currently receiving pharmacologic treatments for mental health issues; having neurological disorders that could affect cognitive processes; and/or failing to provide valid responses to either the pre- or post-assessment surveys. Informed consent was obtained from all participants prior to engaging in any portion of the research.

*Sample size and statistical power*: Sample size determination was primarily based upon practical consideration related to the collaborative nature of this research program. The number of subjects sampled was similar to those reported in other exploratory studies evaluating the cognitive effects of meditation training in college-aged samples (Moore and Malinowski, 2009; Tang et al., 2009). With the assumption of a moderate effect size (d = 0.50), alpha level = 0.05, and a paired samples design, it can be estimated that approximately thirty-four subjects would be needed to obtain 80 percent statistical power. Therefore, the final sample of 40 participants was considered adequate to detect moderate within-subject changes in cognitive and psychological outcomes, although smaller effects may have remained undetected in cognitive functions between pre- and post-meditation periods.

*Meditation program intervention*: A four-week progressive focused-attention meditation program was implemented using attentional meditative techniques derived from yoga-based meditation. For the purposes of this study, yoga-based meditation was operationally defined as contemplative components of yoga aimed at stabilizing attention and facilitating mental concentration. The program did not include *asanas*, vigorous physical exercise, or formal breath-control techniques (*pranayama*), but focused on relaxation, body awareness, and focused attention on breathing. Training was progressive and aimed to gradually increase both practice frequency and attentional stability over time. All sessions lasted 35 min and followed the same sequence: (1) Relaxation phase: participants were guided to relax the body and release physical tension; (2) Body-scan meditation: participants sequentially directed attention toward different body regions; and (3) Focused attention on breathing: participants observed the spontaneous flow of inhalation and exhalation.

Each week of the 4-week program progressed in terms of practice frequency (see table below) and the amount of time spent on the first two segments, allowing the third segment, Focused Attention on Breathing, to increase in length.

Formal practice proceeded following the weekly curriculum described above. Each week participants attended 3 formal practice sessions during weeks 1 and 2, 4 sessions during week 3 and 5 sessions during week 4. Each week at least one formal session occurred in person, whereas other formal sessions could take place in-person or online, at participants’ convenience. The in-person session was used to discuss methods, respond to inquiries, provide individualized support where applicable, practice collectively as well as to promote engagement. The hybrid format was adopted to preserve the contextual and interpersonal dimensions of contemplative practice while allowing flexibility for students’ academic schedules (Dorjee, 2016; Lloyd et al., 2018). Informal practice consisted of individuals engaging in short, frequent mindfulness practices (e.g., mindful breath awareness, body tension scan) outside of their sessions as part of their everyday life.

Attendance at formal practice sessions and weekly self-reported logs (completed by the participant and turned in to the instructor) were used to monitor adherence to both parts of the meditation program. Participants in the study who were evaluated pre and post intervention had to comply with an adherence of over 80% of the practices, which corresponded to 37 participants. Because not all participants completed every questionnaire or cognitive task at both time points, the final sample size varied across outcomes; therefore, the sample size for each analysis is reported in the Results section and corresponding figure legends.

Weekly structure of the intervention.

**Table:** 

Week	Relaxation	Body scan	Breath attention	Sessions/week
Week 1	20 min	10 min	5 min	3
Week 2	10 min	15 min	10 min	3
Week 3	5 min	15 min	15 min	4
Week 4	5 min	10 min	20 min	5

*Practice log*: Each participant maintained weekly practice logs. These logs recorded their adherence to the instructed practices and included minimal self-report data relating to sleep duration, perceived sleep quality, dominant emotional valence during daily life activities and after meditation practices. Other exploratory fields, including practice time, personal goals, and open observations, were not analyzed inferentially but were used pedagogically by the instructor to monitor participants’ progress, address individual difficulties, and adjust the teaching process during the intervention.

## 3 Psychological variables

*Depression, Anxiety and Stress*: Symptoms of depression, anxiety and stress were evaluated by means of the Depression Anxiety Stress Scales-21 (DASS-21) (Lovibond and Lovibond, 1995). DASS-21 is a self-reported instrument consisting of 21 items divided into three sub-scales (depression, anxiety and stress). Each sub-scale includes seven items scored on a four-level Likert-type scale (from 0 = Did not apply to me at all; to 3 = Applied to me very much or most of the time) based on the degree to which each item applies to the individual over the past week. For each subscale, total scores were multiplied by two to obtain values comparable to the full DASS-42 scale. Therefore, higher scores reflect greater symptomatology in each of these areas. The DASS-21 has shown adequate psychometric properties in nonclinical adult samples and has also been validated in Spanish-speaking populations (Daza et al., 2002; Sinclair et al., 2012).

*Anxiety levels*: Anxiety levels were assessed using the State–Trait Anxiety Inventory (STAI) (Spielberger et al., 1983). The STAI assesses both temporary anxiety (state anxiety) and stable tendency towards anxiety (trait anxiety). The inventory includes forty items rated on a four-point Likert scale. A high score indicates greater anxiety. The STAI is a widely used measure of state and trait anxiety and has demonstrated adequate psychometric properties in Spanish-language versions (Spielberger et al., 1983; Virella et al., 1994).

*Mindfulness meta-awareness*: Mindfulness traits were assessed by using the Five Facet Mindfulness Questionnaire (FFMQ) (Baer et al., 2006). The FFMQ evaluates five different aspects of mindfulness: Observing (pay attention to sensations and emotions), Describing (label experiences), Acting with Awareness (act intentionally), Non-Judgment of Inner Experience (view internal experiences without evaluating), and Non-Reactivity to Inner Experience (do not react to internal events). Responses are rated on a five-level Likert-type scale (from 1 = Never or very rarely true; to 5 = Very often or always true). Scores vary from 39 to 195 with higher scores being indicative of increased mindfulness. The FFMQ has shown adequate psychometric properties in terms of factor structure, internal consistency, and construct validity (Baer et al., 2006). Spanish validation studies also support its use in Spanish-speaking populations, and has been validated in Chilean university populations (Cebolla et al., 2012; Schmidt and Vinet, 2015).

*Warwick–Edinburgh Mental Well-being Scale (WEMWBS)*: Psychological well-being was evaluated using the Warwick–Edinburgh Mental Well-being Scale (WEMWBS) (Tennant et al., 2007). The WEMWBS comprises fourteen items referring to positive aspects of mental health based on hedonic and eudaimonic well-being, experienced over the last fortnight. Responses are rated on a five point Likert scale, with higher ratings reflecting improved psychological well-being. The WEMWBS was developed and validated to assess positive mental health and psychological well-being in the general population (Tennant et al., 2007). The Spanish version has demonstrated adequate validity and reliability, and has also been validated in Chilean populations (Carvajal et al., 2015; Castellví et al., 2014).

## 4 Cognitive tasks

*Free recall*: Episodic memory performance was measured using a free recall paradigm. Participants viewed a list of 20 words: 10 with positive emotional content and 10 with negative, all matched for mean syllable length. Each word was displayed individually in sequential order on a monitor. The participant then completed a free recall test twice. On the first test (Immediate), the participant attempted to recall as many words as possible from the list within 30 s after viewing the last word. On the second test (Delayed, 40 min after), the participant attempted to recall as many words as possible from the list 40 min after viewing the last word. For both tests, the participant wrote down every single word that they remembered regardless of the order of those words. To measure memory performance, we counted how many of the original 20 words the participant correctly recalled.

*AOSPAN*: We used a computerized version of the Automated Operation Span Task (AOSPAN; Unsworth et al., 2005) to assess working memory capacity. We utilized PsychoPy (a psychological experimentation library for Python written by Peirce et al., 2019) to program and administer the AOSPAN test. Throughout the experiment, the participant was required to solve a series of arithmetic problems. However, before providing an answer, the participant had to determine if a given number represented the correct answer. A number representing the correct answer appeared on the screen immediately after completing each problem. The participant then indicated whether the number matched their solution. Immediately following completion of the math verification task, a letter appeared. The letter remained visible for a short period of time before disappearing. Once set sizes ranging from 3 to 7 math-letter pairs had been completed, the participant attempted to recall each letter in the order it was presented. There were 75 math-letter trials throughout the entire duration of the test. The participant’s AOSPAN score represented working memory performance and was calculated as the number of letters correctly recalled in the same serial order in which they were presented.

*Sustained Attention to Response Task (SART)*: Sustained attention was assessed using the Sustained Attention to Response Task (SART), a computerized go/no-go paradigm designed to measure attentional stability and response inhibition. The task was implemented using PsychoPy software. Participants were instructed to press the spacebar as quickly as possible whenever a non-target stimulus appeared on the screen (GO trials), and to withhold their response when a designated target stimulus (NoGo trials) was presented. Target trials occurred in approximately 10% of trials. Reaction time (RT) analyses were restricted to correct GO responses (hits), and a plausibility window of 150–1,500 ms was applied to exclude anticipatory and delayed responses. Behavioral outcomes were derived at both the subject level and the trial level. Due to a technical failure during the pre-intervention assessment, RT data were not available for error-related trials (e.g., commission errors). Consequently, RT measures associated with error trials were excluded from all analyses to ensure comparability between pre- and post-intervention conditions; however, accuracy-based measures, including GO accuracy and NoGo accuracy, were retained and analyzed. Primary outcome measures included GO accuracy (proportion of correct responses), mean reaction time for GO trials, reaction time variability, and NoGo accuracy. In addition, trial-level analyses were conducted to examine time-on-task effects and changes in attentional dynamics across the task.

*Statistical analysis*: For quantitative data that were collected through this study, a variety of descriptive and inferential statistical techniques were employed. Means and standard deviations were utilized for continuous variables. Frequencies and percentages were also used for reporting categorical variables. With respect to the application of the appropriate inferential statistics for conducting comparisons of the dependent measure (i.e., change score), the normality of the dependent measure (i.e., change score) was tested via the Shapiro–Wilk test. If normality was demonstrated, then paired-samples Student’s *t*-tests were conducted to assess the differences in the dependent measures across time points. If normality could not be demonstrated, then the Wilcoxon Signed-Rank Test was utilized as an alternative since it is a nonparametric test. Pearson Chi-Square Tests were utilized for assessing categorical variables. Additionally, associations between the continuous variables were examined using Pearson Correlation Coefficients if normality was demonstrated; otherwise, Spearman Rank Correlations were utilized. Furthermore, the magnitude of the treatment effects associated with the intervention conditions were quantified using Cohen’s d for paired samples; this effect size metric represents the mean change in each participant’s dependent measures from one time point to another relative to the pooled standard deviation of those dependent measures. In addition, effect size interpretations of the findings were made utilizing established conventions (e.g., small: 0.20; medium: 0.50; large: 0.80). Missing data were not imputed; analyses were conducted using available complete pre–post data for each outcome.

For SART outcomes, both subject-level and trial-level assessments were completed. Subject-level evaluations of the SART data consisted of paired-sample t-test and Wilcoxon Signed-Rank Test evaluations of the pre-post changes in SART performance. Both t-test and Wilcoxon Signed-Rank Test evaluations were conducted so as to demonstrate the robustness of the results due to possible nonnormal distributions of the proportion data. In order to quantify the magnitude of these changes, Cohen’s dz for paired sample comparisons and rank-biserial correlations for nonparametric tests were computed. Finally, to adjust for potential Type I errors resulting from conducting multiple comparisons across SART outcomes, *p*-values were adjusted using Holm Procedure and estimates of False Discovery Rate (FDR) were also generated using Benjamini-Hochberg Method. Due to the limited sample size and the upper limit nature of proportion data, both parametric and nonparametric evaluation metrics were computed for descriptive purposes. Ultimately, however, all primary inferences regarding trial-level SART performance were drawn from trial-level Mixed Models. Trial-Level Evaluations - Mixed Effects Models were fit at the trial level to accommodate for interparticipant variability and dependency. Reaction Time Data - Correct GO Trials Reaction times from correct GO trials were analyzed using Linear Mixed Model (LMM) methodology with time (Pre vs. Post) as a fixed factor and random intercepts for each participant. Secondary model specifications included inclusion of z-scored trial index (i.e., time-on-task) and its interaction with time to assess changes in attentional processing over time. GO Accuracy - Accuracies for GO trials were analyzed using Generalized Linear Mixed Model (GLMM) methodology with Binomial Distribution and Logit Link. Fixed Effects Regression Coefficient with 95% Confidence Intervals for Fixed Effects are reported; Odds Ratios are also reported for Logistic Models. Unfortunately, reaction time data for Error Trials were unavailable during initial baseline data collection; thus, reaction time data for error trials were removed from all analyses so as to allow for valid Pre-Post Comparisons. All primary analyses were performed utilizing GraphPad Prism 6 software and all secondary analyses were conducted using R Studio software.

*Ethical approval*: All participants provided written informed consent prior to participation. The study protocol was approved by the Bioethics Committee of Universidad Santo Tomás (Approval number: 42–2023). All procedures were conducted in accordance with the Declaration of Helsinki for research involving human participants.

## 5 Results

### 5.1 Sample characterization during the intervention

**Figure 2:**
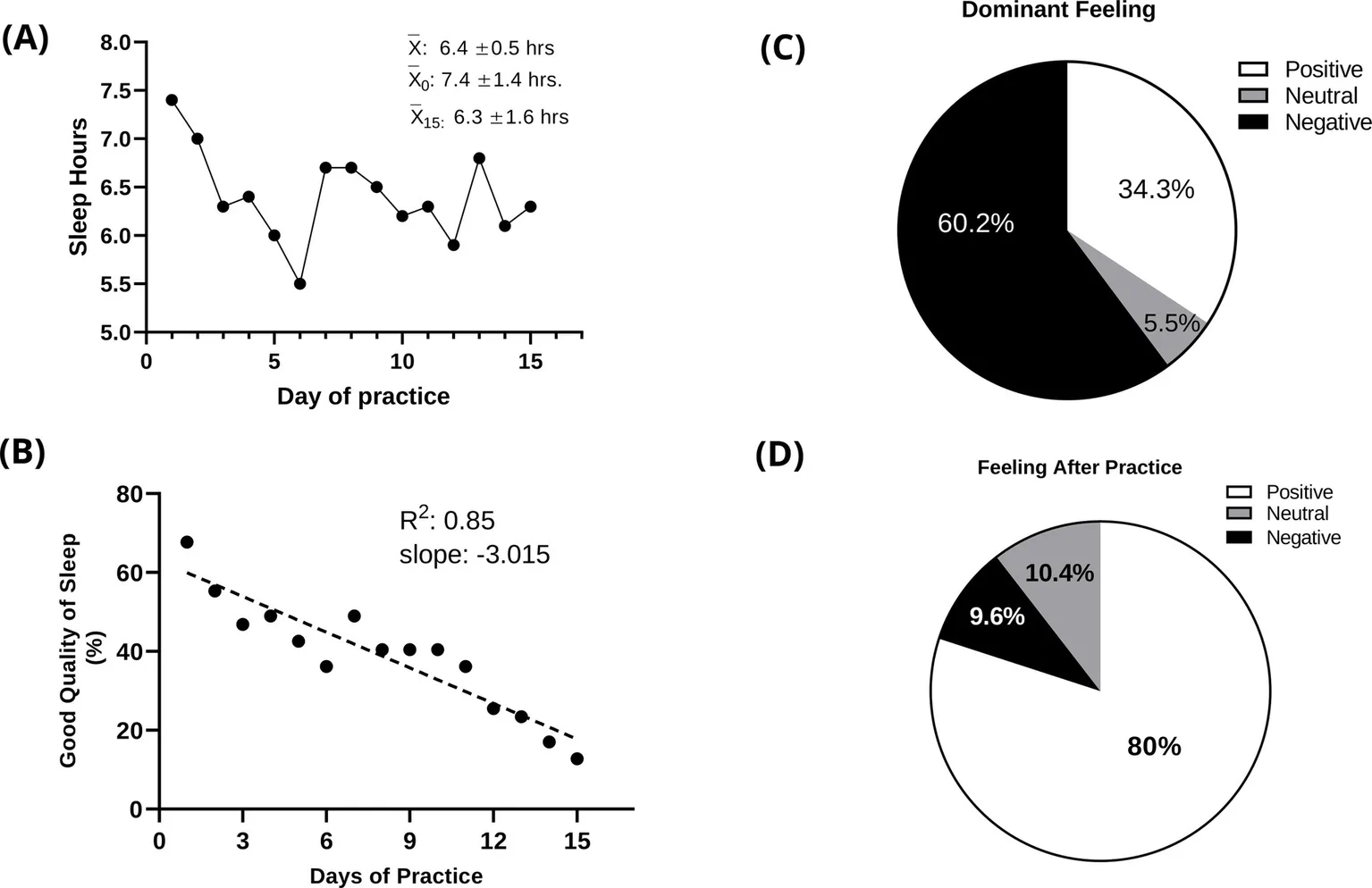
Sample characterization during the intervention period. **(A)** Mean hours of sleep per night reported by participants across the six-week intervention period, showing a declining trend over time. **(B)** Proportion of participants reporting “good” sleep quality across the intervention period, indicating a trend toward decreased subjective sleep quality. **(C)** Distribution of the predominant emotional valence (positive, neutral, or negative) reported during daily activities throughout the intervention, showing a predominance of negatively valenced cognitive-emotional content. **(D)** Distribution of overall emotional valence reported at the end of each meditation session, showing a shift toward positive and neutral valence relative to the daily activity pattern shown in **(C)**.

The sample consisted of 40 undergraduate medical students, with a mean age of 23.9 ± 1.9 years. Regarding sex distribution, 76% were women and 24% were men. Participants were distributed across the first 4 years of medical training: 8 students were in the first year (20.0%), 5 in the second year (12.5%), 23 in the third year (57.5%), and 4 in the fourth year (10.0%). The intervention was conducted during the final 6 weeks of the academic semester. Although academic stress was not assessed continuously across the semester, weekly practice logs provided descriptive information about sleep duration, perceived sleep quality, and emotional valence during the intervention period. These descriptive data showed a decrease in sleep duration across the study period (Figure 2A), together with a trend toward lower perceived sleep quality (Figure 2B).

In addition, there was a prevalence of negative valence of cognitive-emotional content during daily activities in participants throughout the experiment (Figure 2C). After meditation practice, however, the distribution of reported valence shifted toward more positive and neutral states (Figure 2D).

Overall, these descriptive results characterize the intervention period as one marked by reduced sleep and a predominance of negatively valenced daily emotional content. These data provide contextual background for interpreting the psychological and cognitive outcomes reported below.

### 5.2 Psychological outcomes

We assessed pre-to-post changes in students’ anxiety, depression, and stress during the intervention period.

**Figure 3:**
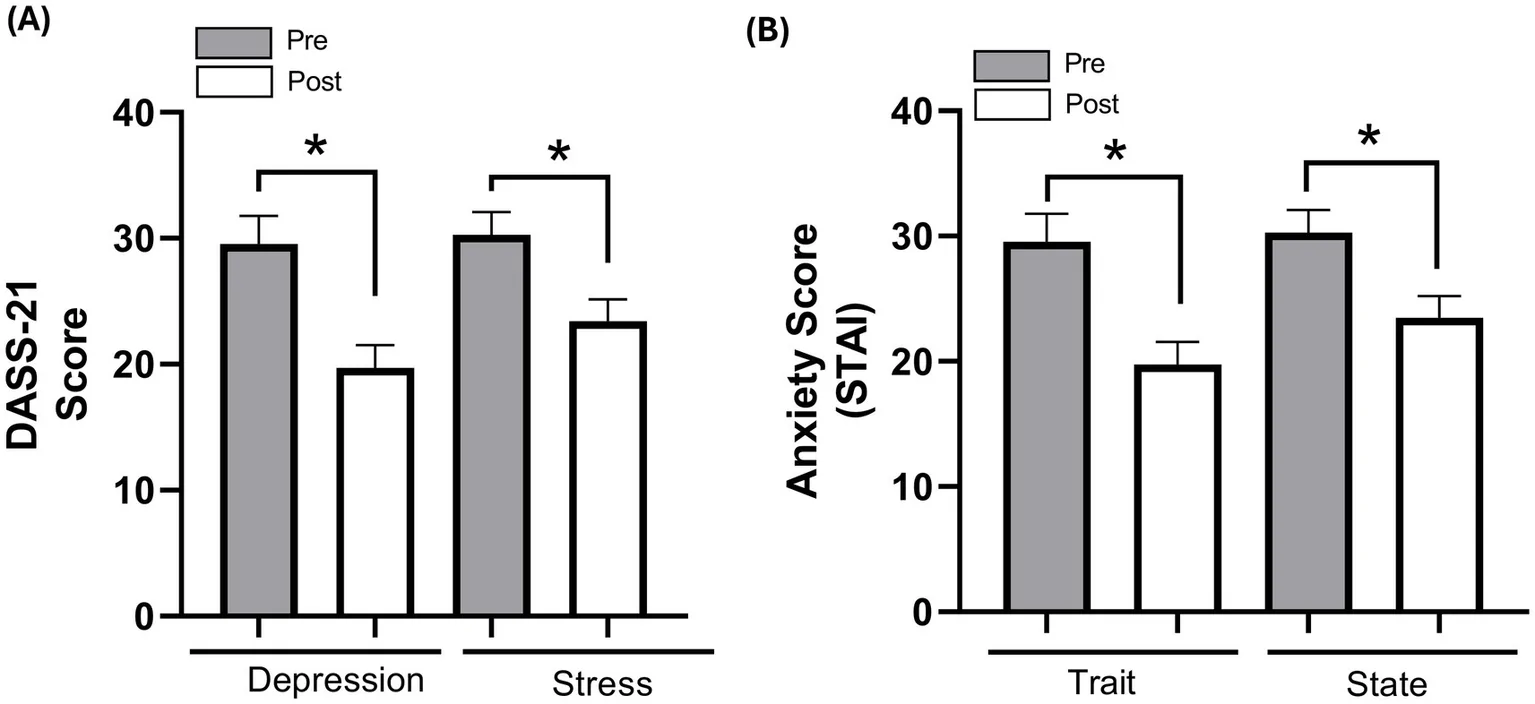
Yoga-based meditation was associated with reductions in anxiety, depression, and stress in medical students. **(A)** Mean DASS-21 depression and stress scores before (Pre) and after (Post) the intervention (*n* = 29). Significant pre-to-post decreases were observed for depression [*t*(28) = −3.83, *p* < 0.001, d = 0.71] and stress [t(28) = −3.19, *p* = 0.004, d = 0.59]. **(B)** Mean STAI State and Trait anxiety scores before and after the intervention (State Anxiety n = 29; Trait Anxiety n = 27). Significant reductions were observed for both State Anxiety [t(28) = −3.19, p = 0.004, d = 0.59] and Trait Anxiety [t(26) = −3.61, *p* = 0.001, d = 0.69]. Error bars represent standard error of the mean. DASS-21, Depression Anxiety Stress Scales–21; STAI, State–Trait Anxiety Inventory.

Students with complete DASS-21 data (*n* = 29) exhibited a significant decrease in depressive symptomatology as described in Figure 3A (pre-mean = 29.6 ± 11.98; post-mean = 19.7 ± 9.73; Shapiro–Wilk *p* = 0.66; t(28) = −3.83, *p* < 0.001; Cohen’s d = 0.71), as well as stress (pre-mean = 30.3 ± 9.74; post-mean = 23.4 ± 9.39; Shapiro–Wilk *p* = 0.07; t(28) = −3.19, *p* = 0.004; Cohen’s d = 0.59).

Additionally, among participants with complete STAI data, trait anxiety (*n* = 27) significantly decreased from pre- to post-intervention (pre-mean = 29.7 ± 12.2; post-mean = 19.8 ± 10.1; Shapiro–Wilk *p* = 0.62; t(26) = −3.61, *p* = 0.001; Cohen’s d = 0.69). A similar reduction was observed for state anxiety (pre-mean = 30.3 ± 9.75; post-mean = 23.5 ± 9.37; Shapiro–Wilk *p* = 0.08; t(28) = −3.19, *p* = 0.004; Cohen’s d = 0.59) (Figure 3B).

Collectively, these results indicate that the meditation intervention was associated with reductions in self-reported negative affective states during the intervention period.

### 5.3 Well-being and mindfulness meta-awareness

In addition to symptoms of depression, anxiety, and stress, we assessed pre-to-post changes in psychological well-being and mindfulness-related traits. Psychological well-being was measured using the WEMWBS, whereas mindfulness-related traits were assessed using the FFMQ.

**Figure 4:**
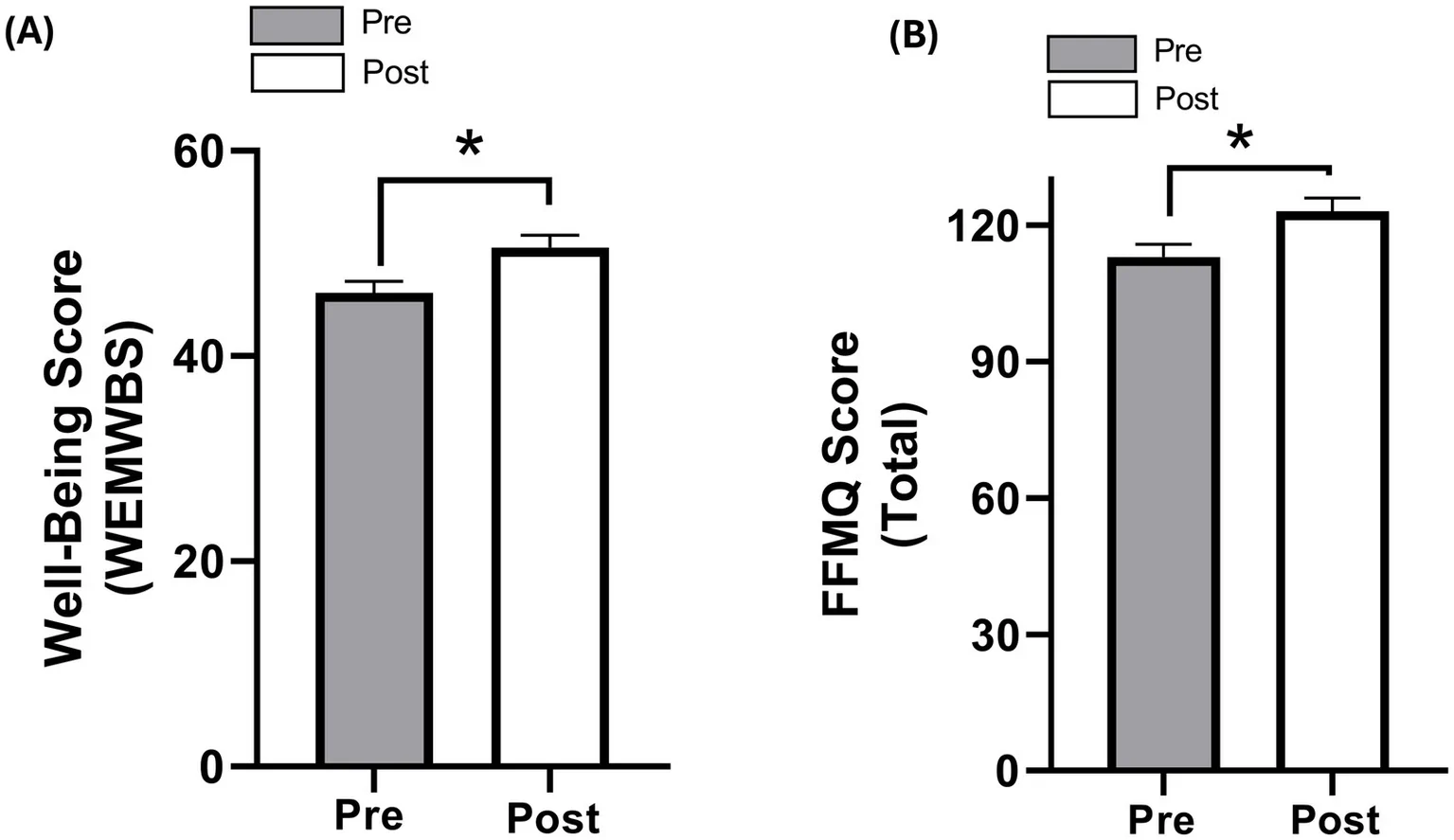
Yoga-based meditation training is associated with improvements in psychological well-being and global mindfulness. **(A)** Mean WEMWBS total scores before (Pre) and after (Post) the intervention (*n* = 37). A significant increase in well-being was observed post-intervention [*t*(36) = 2.63, *p* = 0.013, *d* = 0.43]. **(B)** Mean total FFMQ scores before and after the intervention (*n* = 31). A significant increase in global mindfulness was observed post-intervention [*t*(30) = 3.57, *p* = 0.001, *d* = 0.64]. No significant differences were found for individual FFMQ subscales (see Supplementary materials). Error bars represent standard error of the mean. WEMWBS, Warwick-Edinburgh Mental Well-being Scale; FFMQ, Five Facet Mindfulness Questionnaire.

In this context, participants with complete WEMWBS data (n = 37) showed an increase in their level of mental wellness after completing the study (46.19 ± 6.71 vs. 50.59 ± 7.05; Shapiro–Wilk *p* = 0.55; t(36) = 2.63, *p* = 0.013, d = 0.43) (Figure 4A).

Similarly, participants with complete FFMQ data (*n* = 31) demonstrated a significant increase in global mindfulness after participating in the study (113.26 ± 16.05 vs. 122.84 ± 16.33; Shapiro–Wilk *p* = 0.11; t(30) = 3.57, *p* = 0.001, d = 0.64). However, no significant differences were found when comparing individual FFMQ subscales, including observing, describing, acting with awareness, non-judging, and non-reactivity (Figure 4B).

Together, these findings show significant pre-to-post increases in psychological well-being and global mindfulness scores.

### 5.4 Working memory

**Figure 5:**
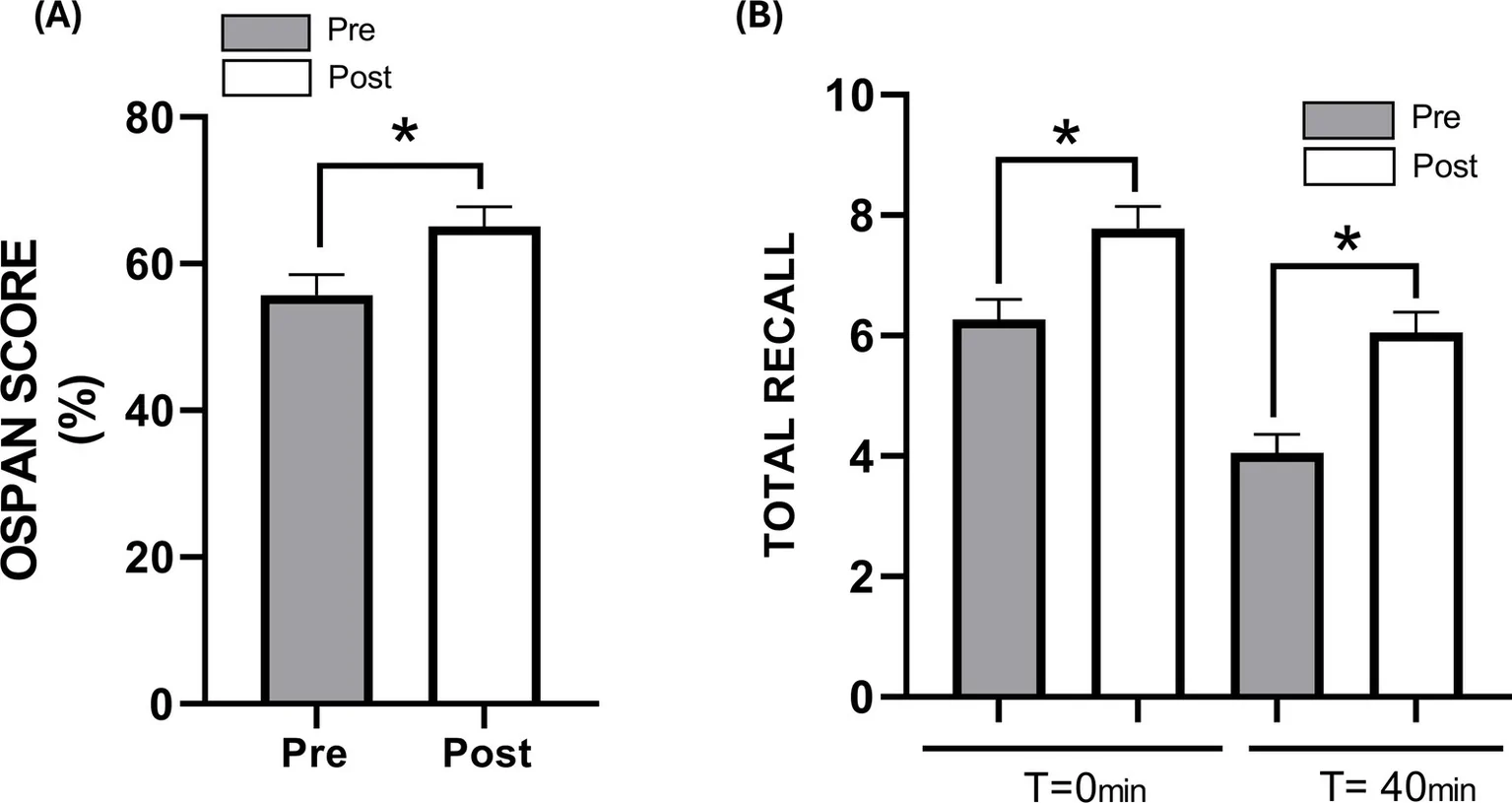
Pre-to-post changes in working memory and episodic memory after yoga-based meditation. **(A)** Mean AOSPAN scores before (Pre) and after (Post) the intervention (*n* = 37). A significant increase in working memory capacity was observed post-intervention [*t*(36) = 2.62, *p* = 0.013, *d* = 0.43]. **(B)** Mean episodic memory performance for immediate recall (T0) and delayed recall (T40) before and after the intervention (*n* = 34). Significant improvements were observed for both immediate recall [*t*(33) = 3.97, *p* < 0.001, *d* = 0.68] *and* delayed recall [Wilcoxon Z = 3.67, *p* < 0.001, *d* = 0.81]. Error bars represent standard error of the mean. AOSPAN, Automated Operation Span Task; T0, immediate recall; T40, delayed recall at 40 min.

Working memory was assessed using the AOSPAN task. Participants with complete AOSPAN data (*n* = 37) showed greater working memory performance after the intervention (55.68 ± 17.14 vs. 65.11 ± 16.18; Shapiro–Wilk *p* = 0.48; t(36) = 2.62, *p* = 0.013, d = 0.43) (Figure 5A). This result indicates a significant pre-to-post increase in AOSPAN performance.

### 5.5 Episodic memory performance

Episodic memory performance was assessed using immediate and delayed free recall. We examined whether recall performance changed from pre- to post-intervention.

Among participants with complete free recall data (*n* = 34), immediate total recall at T0 improved post-intervention (6.26 ± 2.14 vs. 7.68 ± 2.23; Shapiro–Wilk *p* = 0.11; t(33) = 3.97, *p* < 0.001, d = 0.68) (Figure 5B).

Delayed total recall at T40 also improved post-intervention in the same sample (*n* = 34) (4.24 ± 2.05 vs. 5.85 ± 1.94; Shapiro–Wilk *p* = 0.04; Wilcoxon Z = 3.67, *p* < 0.001, d = 0.81) (Figure 5B).

We also compared recall of positive and negative words and tested whether emotional valence modulated performance to detect potential biases in the transfer from working memory to long-term episodic memory. Recall increased similarly for both word types at both time points (T = 0 and T = 40), with no evidence of a valence-specific pattern in the descriptive comparison (see Supplementary Figure 1).

In conclusion, immediate and delayed recall both increased from pre- to post-intervention, and this pattern was observed across positive and negative word categories.

### 5.6 Sustained attention

**Figure 6:**
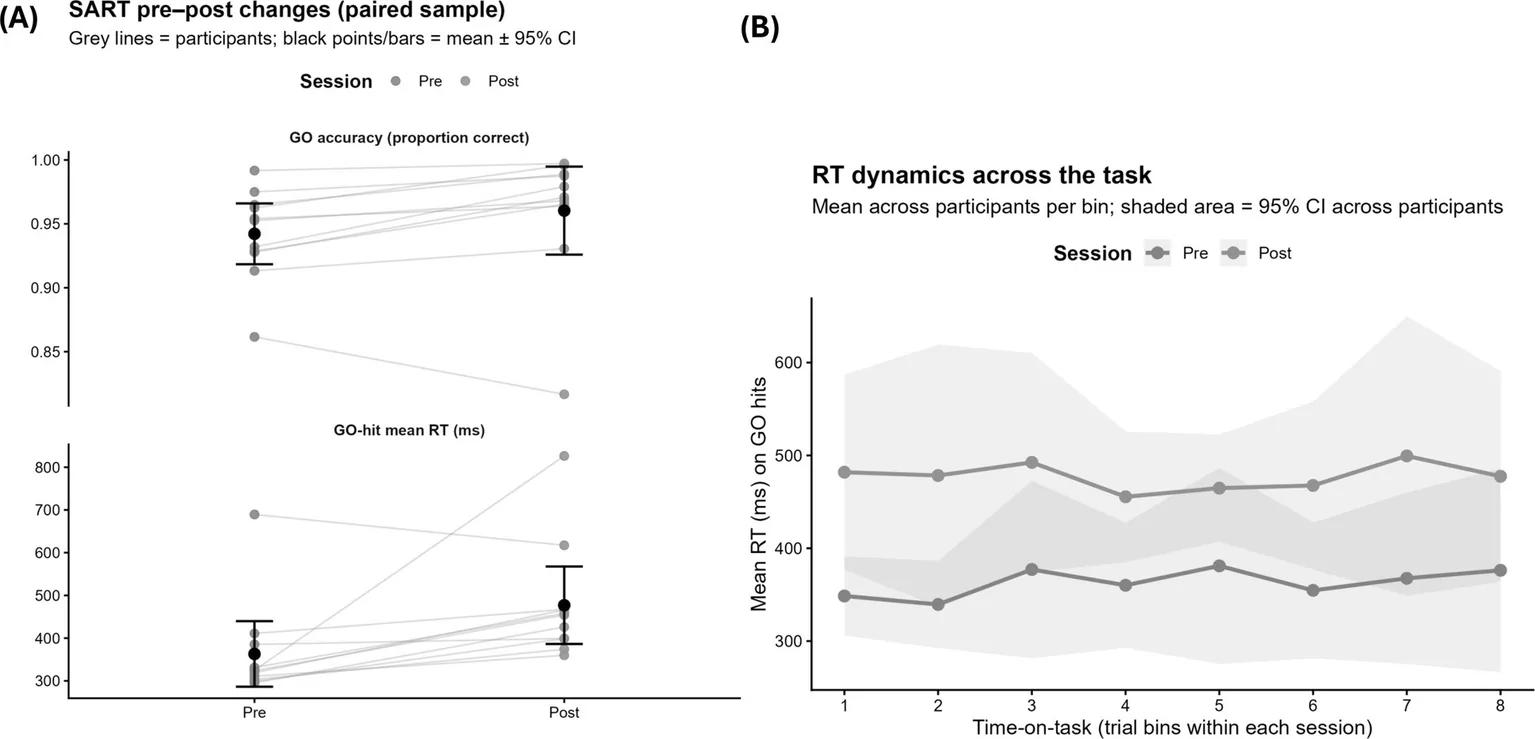
SART performance across sessions. **(A)** Pre-to-post changes in GO accuracy (proportion correct; top panel) and GO-hit mean reaction time (RT; ms; bottom panel) in the paired sample (*n* = 11). Each colored point represents an individual participant; grey lines connect pre and post values for the same participant. Black points and error bars indicate the group mean ± 95% confidence interval. Green = Pre session; Orange = Post session. **(B)** RT dynamics across the task. Mean RT (ms) on correct GO trials (hits) plotted across eight equal-sized time-on-task bins computed within each session (Pre, Post) for the paired sample (*n* = 11). Points represent the group mean per bin; lines connect bin means within each session. Shaded bands indicate 95% confidence intervals across participants. A significant time × trial-bin interaction (*p* < *0.001*) indicated that RT dynamics differed between sessions, with post-session RTs remaining consistently elevated relative to pre-session across the task. SART, Sustained Attention to Response Task; RT, reaction time; LMM, linear mixed model.

Sustained attention was assessed using the SART. Because baseline (pre) RTs were not available for error-related trials due to a technical failure in the stimulus presentation/response logging system, commission/omission error metrics and any RT-on-error outcomes were not included in the reported analyses. We therefore report (a) GO accuracy, (b) GO RT measures computed on correct GO responses (hits), and (c) NoGo accuracy. In the paired sample (*n* = 11), GO accuracy increased from pre (M = 0.942) to post (M = 0.960), t(10) = 2.40, *p* = 0.038, dz. = 0.72, 95% CI [0.04, 1.38] (Table 1). A consistent pattern was observed in the Wilcoxon signed-rank test, V = 56, *p* = 0.045. For response speed, GO-hit mean RT increased from pre (M = 363.06 ms) to post (M = 477.03 ms), t(10) = 2.63, *p* = 0.025, dz. = 0.79, 95% CI [0.10, 1.46]; Wilcoxon: V = 61, *p* = 0.014 (Table 1). GO-hit median RT showed a similar direction but did not reach the conventional threshold (*p* = 0.056). NoGo accuracy did not differ between sessions (Table 1) (Figure 6A). When correcting for multiple comparisons across the retained paired outcomes (five outcomes), the paired effects did not remain below 0.05 (see Supplementary Table S1 for adjusted *p* values). For transparency, we therefore treat the paired results as supportive/descriptive and rely on the trial-level mixed-effects models for the primary inferential evidence.

**Table 1:** Paired pre–post outcomes (main sample; *n* = 11).

Variable	*n* pre mean	Post mean	∆*M* (post-pre)	Paired *t*	95% CI (∆)	*d_z_* [95% CI]	Wilcoxon	*r_rb_*
GO accuracy	110.942	0.96	0.018	*t*(10) = 2.40, *p* = 0.038	[0.001, 0.035]	0.72 [0.04, 1.38]	V = 56, *p* = 0.045	0.7
GO RT median	11367.54	442.36	74.81	*t*(10) = 0.86, *p* = 0.409	[−118.77, 268.40]	0.26 [−0.35, 0.85]	V = 55, *p* = 0.056	0.67
GO RT mean	11363.06	477.03	113.97	*t*(10) = 2.63, *p* = 0.025	[17.50, 210.45]	0.79 [0.10, 1.46]	V = 61, *p* = 0.014	0.85
GO RT SD	11127.86	154.56	26.7	*t*(10) = 1.90, *p* = 0.086	[−4.59, 57.98]	0.57 [−0.08, 1.20]	V = 54, *p* = 0.068	0.64
NoGo accuracy	110.499	0.498	−0.002	*t*(10) = −0.05, *p* = 0.958	[−0.064, 0.061]	−0.02 [−0.61, 0.58]	V = 32, *p* = 0.965	−0.03

RT outcomes were computed on correct GO responses (hits) using the cleaned RT window (150–1,500 ms). r_rb_ is the rank-biserial correlation computed from the Wilcoxon V statistic.

Trial-level modeling converged with the paired patterns and provided strong evidence for session differences. In the RT LMM (GO-hit trials; random intercepts for participants), RTs were slower at post than pre (B = 113.72 ms, 95% CI [108.04, 119.40]), t(13, 557) = 39.24, *p* < 0.001 (Table 2). In the time-on-task LMM, the main effect of time remained significant (B = 107.77 ms, *p* < 0.001), and there were significant effects of trial index (B = 5.71 ms, *p* = 0.002) and a time × trial interaction (B = −24.59 ms, *p* < 0.001) (Figure 6B) indicating that RT dynamics across the task differed between pre and post (Table 2).

**Table 2:** Fixed effects from Linear Mixed Models (RT) and Generalized Linear Mixed Models (Accuracy).

Model	Effect	*B*	*SE*	95% CI	Test	*p*	OR	95% CI (OR)
RT LMM (basic)	Intercept	365.45	30.40	[305.86, 425.04]	*t*(11.04) = 12.02	< 0.001	–	–
	Time (post vs. pre)	113.72	2.90	[108.04, 119.40]	*t*(13557) = 39.24	< 0.001	–	–
RT LMM (time-on-task)	Intercept	362.66	30.36	[303.15, 422.17]	*t*(11.06) = 11.94	< 0.001	–	–
	Time (post vs. pre)	107.77	3.43	[101.04, 114.50]	*t*(13556) = 31.39	< 0.001	–	–
	Trial*_z_*	5.71	1.85	[2.08, 9.33]	*t*(13556) = 3.09	0.002	–	–
	Time *×* Trial*_z_*	−24.59	3.97	[−32.37, −16.80]	*t*(13555) = −6.19	< 0.001	–	–
GO Accuracy GLMM	Intercept	3.107	0.265	[2.588, 3.625]	*z* = 11.75	< 0.001	22.35	[13.31, 37.54]
	Time (post vs. pre)	0.395	0.077	[0.244, 0.547]	*z* = 5.12	< 0.001	1.48	[1.28, 1.73]

B, Unstandardized beta estimate; SE, Standard Error; OR, Odds Ratio. For the LMMs, the degrees of freedom were estimated using the Satterthwaite method. Trial_z_ refers to the z-scored trial index to assess time-on-task effects.

In the GO accuracy GLMM (binomial-logit; random intercepts for participants), accuracy was higher at post (log-odds B = 0.395), OR = 1.48, 95% CI [1.28, 1.73], z = 5.12, *p* < 0.001 (Table 2).

Collectively, these findings show higher GO accuracy and slower GO-hit RTs after the intervention, consistent with a more cautious response pattern on the SART.

## 6 Discussion

The present pilot study found pre-to-post improvements in several cognitive and psychological indicators related to self-regulation after participation in a yoga-based meditation program. Given the single-group pre–post design, these findings should be interpreted as preliminary associations rather than causal effects. Still, the pattern is compatible with the possibility that a short, structured contemplative intervention may be associated with changes across emotional/affective and cognitive/behavioral domains. This is relevant because self-regulation is unlikely to depend on a single psychological process. It appears to involve the coordination of affective regulation, attentional monitoring, cognitive control, and the way individuals relate to their own internal experience. In this sense, the observed reductions in anxiety, depression, and stress, together with increases in well-being and global mindfulness, are broadly aligned with previous studies suggesting that contemplative interventions may help reduce distress and support psychological functioning in medical students and other high-demand populations (Aljuwaiser et al., 2024; Fazia et al., 2023; Hathaisaard et al., 2022; Sperling et al., 2023; Wang et al., 2024).

The cognitive results add a further layer to this interpretation. Participants showed higher AOSPAN scores after the intervention, which may indicate better working memory task performance. Free recall also improved in both immediate and delayed conditions. Importantly, this pattern was observed for both positive and negative words, suggesting that the effect was not simply driven by a valence-specific recall bias. Still, these findings should be interpreted carefully. The study did not isolate specific memory mechanisms, and the design does not allow us to determine whether the changes were due to meditation itself, repeated testing, or other contextual factors. Even so, the pattern is compatible with recent evidence suggesting that contemplative interventions may be associated with changes in working memory, attention, and recall accuracy (Jong et al., 2025; Whitfield et al., 2021; Zainal and Newman, 2023).

The SART results also suggest changes in response dynamics after the intervention. Participants showed higher GO accuracy together with slower GO-hit reaction times. Rather than indicating a direct improvement in sustained attention or a reduction in mind-wandering, this pattern is better understood as a shift toward a more cautious response strategy. The trial-level analyses further suggested that response-time dynamics differed between pre- and post-intervention assessments. However, mind-wandering was not directly measured through experience sampling, thought probes, or trial-by-trial self-report. For this reason, the SART results should be interpreted as behavioral evidence of altered response dynamics, not as direct evidence of changes in spontaneous thought.

Although the present study did not measure neural activity, the overall pattern of findings can be situated within current neurocognitive models of meditation and yoga-based practices. Previous work suggests that contemplative training may engage both top-down regulatory mechanisms, such as attentional control, executive monitoring, and meta-awareness, and bottom-up processes related to interoceptive, autonomic, and bodily feedback (Dahl et al., 2015; Gard et al., 2014; Lutz et al., 2008; Schmalzl et al., 2015; Tang et al., 2015). These processes are also compatible with models involving the default mode, frontoparietal, and salience networks, which have been linked to self-referential processing, attentional control, and interoceptive awareness (Calderone et al., 2024; Sezer et al., 2022; Tang et al., 2015). These neural models provide a useful theoretical context, but they should not be taken as evidence that such neural mechanisms were directly modified in the present sample.

A central point of the study is that the intervention was not designed to represent the whole yoga system. Classical yoga, particularly in the eight-limbed framework of Ashtanga Yoga, includes ethical, postural, respiratory, sensory, attentional, meditative, and absorptive dimensions. Thus, excluding asanas and formal pranayama does not mean that these practices are being treated as non-contemplative. The decision was methodological: we focused on the more internal attentional dimensions of practice, especially concentration and meditation, in order to reduce heterogeneity and better isolate the possible contribution of progressive attentional training to self-regulation. This distinction matters because yoga-based interventions vary widely. Some programs emphasize postures, others breathing practices, relaxation, ethical-philosophical training, or meditation; these components may share certain regulatory pathways, but they may also engage partly different processes (Gard et al., 2014; Schmalzl et al., 2015). In that sense, compared with standardized mindfulness-based programs such as MBSR or MBCT, the present intervention was shorter, more specifically oriented toward attentional stabilization, and less dependent on broader psychoeducational content. The findings should therefore be understood as preliminary evidence related to this specific attention-centered yoga-based meditation protocol, rather than as evidence for yoga or mindfulness-based interventions in general.

This pattern of results can also be conceptualized within the S-ART framework, which links self-awareness, self-regulation, and self-transcendence with contemplative practice (Vago and Silbersweig, 2012). From this point of view, the observed reductions in negative affect, increases in well-being and global mindfulness, and improvements in cognitive task performance may reflect a broader change in the mind’s regulatory capacity. One possible interpretation is that meditation does not only reduce psychological distress; it may also modify how individuals monitor and respond to internal experience. This idea is close to contemplative models of decentering (Dahl et al., 2015; Poletti et al., 2021; Quek et al., 2021), in which mental states are perceived as transient events rather than static features of identity. However, this remains a theoretical interpretation. The present study did not directly assess moment-to-moment meta-awareness, decentering, or phenomenological changes in self-experience.

These findings may be particularly relevant in medical education. Medical students face sustained cognitive demands, emotionally intense learning environments, and frequent psychological distress. In that context, interventions that target self-regulation may be useful not only because they reduce symptoms, but because they may support cognitive processes that are directly relevant to learning. Working memory and episodic memory contribute to knowledge acquisition and clinical reasoning, while sustained attention is needed in situations of high cognitive load (Bailey et al., 2020; Ma et al., 2021; Namias and Huff, 2024; Pragya et al., 2021). A brief meditation-based program is not a substitute for structural changes in medical education, but it may offer a low-cost and scalable complement to broader strategies aimed at student well-being and academic functioning.

From an implementation perspective, future research should examine whether these practices can realistically be incorporated into medical curricula. In Latin America and South American countries, structured self-care and contemplative programs remain insufficiently integrated into medical education, despite growing concern about student well-being. Recent evidence suggests that contemplative-based interventions for medical students are promising, but their implementation remains heterogeneous in duration, format, delivery mode, and curricular integration (Kaisti et al., 2023; Wan et al., 2024). Larger multicenter studies are therefore needed, not only to evaluate effectiveness, but also to assess curricular feasibility, protected time for practice, instructor training, institutional support, and long-term sustainability.

Several limitations should be noted. First, the study used a single-group pre–post pilot design and did not include an active or waitlist control group; therefore, causal interpretations should be made cautiously, since practice effects, expectancy effects, regression toward the mean, and contextual factors cannot be ruled out. Second, although the study was designed to detect moderate within-subject effects, the sample size was limited and varied across outcomes due to incomplete data. Third, we did not assess direct neural activity or mind-wandering using experience-sampling, thought probes, or trial-by-trial self-report. Future studies should use controlled experimental designs, longitudinal follow-up, and multimethod assessments, including neuroimaging, psychophysiology, experience-sampling, and active comparison conditions.

Overall, this pilot study suggests that yoga-based meditation was associated with pre-to-post improvements in psychological well-being, affective symptoms, mindfulness, and cognitive task performance in medical students. The findings are compatible with the idea that attention-centered contemplative training may support self-regulation through coordinated psychological and cognitive processes. At the same time, controlled studies are needed to determine whether these changes are specific to meditation practice and to clarify the mechanisms through which yoga-based meditation may influence self-regulation in medical education.

## Data Availability

The original contributions presented in the study are included in the article/Supplementary material, further inquiries can be directed to the corresponding author.
