# Supplementary material for: Yoga-based meditation improves self-regulation and well-being in medical students: a pilot study
Source: Front Hum Neurosci. 2026 Aug 12;20:1844429. doi: 10.3389/fnhum.2026.1844429 (PMC13506737; doi:10.3389/fnhum.2026.1844429)
Supplement: Supplementary file 1 [file Table_1.docx]

Supplementary 1: Comparison of unadjusted and adjusted *p*-values (Holm and FDR)

Paired *t*-test (*p*-values)	Wilcoxon Signed-Rank (*p*-values)

| Outcome | Unadj. | Holm | FDR |  | Unadj. | Holm | FDR |  |
| --- | --- | --- | --- | --- | --- | --- | --- | --- |
| GO accuracy | .038 | .150 | .094 |  | .045 | .182 | .085 |  |
| GO RT median | .409 | .819 | .512 |  | .056 | .182 | .085 |  |
| GO RT mean | .025 | .125 | .094 |  | .014 | .072 | .072 |  |
| GO RT SD | .086 | .259 | .144 |  | .068 | .182 | .085 |  |
| NoGo accuracy | .958 | .958 | .958 |  | .965 | .965 | .965 |  |

*Note.* FDR = False Discovery Rate (Benjamini-Hochberg correction). Unadj. = Unadjusted *p*-value.
